# New sensing platform of poly(ester-urethane)urea doped with gold nanoparticles for rapid detection of mercury ions in fish tissue†

**DOI:** 10.1039/d1ra03693a

**Published:** 2021-09-28

**Authors:** Hany Abd El-Raheem, Rabeay Y. A. Hassan, Rehab Khaled, Ahmed Farghali, Ibrahim M. El-Sherbiny

**Affiliations:** Center of Materials Sciences, Zewail City of Science and Technology October Gardens, 6th of October City 12578 Giza Egypt ielsherbiny@zewailcity.edu.eg ryounes@zewailcity.edu.eg; Applied Organic Chemistry Department, National Research Centre (NRC) Dokki 12622 Giza Egypt; Materials Science and Nanotechnology Department, Faculty of Postgraduate Studies for Advanced Sciences, Beni-Suef University Beni-Suef Egypt; Chemistry Department, Faculty of Science, Beni-Suef University Beni-Suef Egypt

## Abstract

A new electrochemical sensor has been fabricated based on the *in situ* synthesis of poly(ester-urethane) urea (PUU) doped with gold nanoparticles (AuNPs), and the obtained composite materials (PUU/AuNPs) were used as a new sensing platform for highly sensitive and selective detection of mercury(II) ions in fish tissue. PUU was synthesized and fully characterized by XRD, TGA, DSC, and FTIR to analyze the chemical structure, thermal stability, and morphological properties. As a polymeric structure, the PUU consists of urethane and urea groups that possess pronounced binding abilities to Hg^2+^ ions. SEM-EDX was carried out to confirm this kind of interaction. Using ferricyanide as the redox probe, PUU alone exhibited weak electrochemical signals due to its low electrical conductivity. Therefore, a new series of nanocomposites of PUU with different nanostructured materials were applied, and their electrochemical performances were evaluated. Among these materials, the PUU/AuNP-modified electrode showed high voltammetric signals towards Hg^2+^. Consequently, the parameters affecting the performance of the assay, such as electrode composition, scan rate, and sensing time, as well as the effect of electrolyte and pH were studied and optimized. The sensor showed a linear range of 5 ng mL^−1^ to 155 ng mL^−1^ with the regression coefficient *R*^2^ = 0.986, while the calculated values of the limit of detection (LOD) and limit of quantification (LOQ) were 0.235 ng mL^−1^ and 0.710 ng mL^−1^, respectively. In terms of cross reactivity testing, the sensor exhibited a high selectivity against heavy metals which are commonly determined in seafood (Cd^2+^, Pb^2+^, As^3+^, Cr^3+^, Mg^2+^, and Cu^2+^). For real applications, total Hg^2+^ ions in fish tissue were determined with very high recovery and no prior complicated treatments.

## Abstract 2

A new electrochemical sensor based on poly(ester-urethane) urea doped with gold nanoparticles (PUU/AuNPs) for highly sensitive and selective detection of mercury(ii) ions in fish tissue.

## Graphical abstract

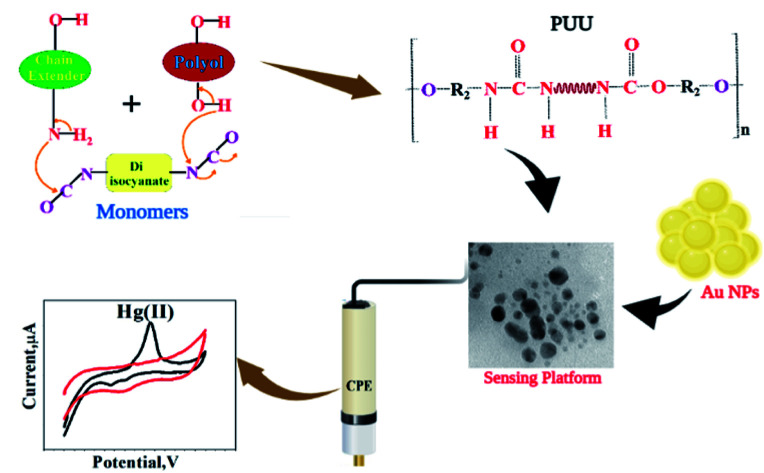

## Introduction

Liquid mercury was found historically in Egyptian tombs from 1500 BC. It was isolated by the ancient Egyptians and the Chinese from the mineral cinnabar (mercuric sulfide).^1^ Mercury, a heavy, silvery-white liquid metal, remains a highly toxic element and the most worrying pollutant at the global scale as it exists inclusively in the ambient atmosphere, water, soil, and numerous bioregions.^2^ Inhalation of mercury vapor leads to harmful effects on nervous, digestive and immune systems, and the lungs and kidneys. The inorganic salts of mercury are corrosive to the skin, eyes, and gastrointestinal tract, and may induce kidney toxicity if ingested.^3–5^ Even at low concentrations, mercury is not only a threat to human health, but also to animals, microorganisms, and plants.^6,7^ An inorganic species Hg^2+^, is one of the common and most stable forms of mercury toxins, and has high tendency to be bio-accumulated in living organisms throughout the food chains, which leads to high concentrations at the top of the aquatic food chain.^8^ Fish and other seafood products can absorb this toxic heavy metal in the gastrointestinal tract, and is then transformed to people who consume amounts of these food products.^9,10^ Consequently, symptoms of health problems include skin rashes and dermatitis, mood swings, memory loss, mental disturbances, muscle weakness, changes in nerve responses, performance deficits on tests of cognitive function.^11^ Thus, due to these health risks, it is very crucial to detect Hg^2+^ with simple and efficient methods to mitigate such possible risks. Mercury can be quantified by several analytical techniques, such as inductively coupled plasma atomic emission spectrometry (ICP-AES),^12^ inductively coupled plasma-mass spectrometry (ICP-MS),^3^ atomic absorption spectroscopy (AAS),^13^ High performance liquid chromatography-cold vapor-inductively coupled plasma mass spectrometry (HPLC/CV/ICP/MS),^14^ and flame atomic absorption spectrophotometry (FAAS).^15^ These methods deliver high sensitivity, low detection, and wide linearity range with low limit of detection. However, they do not meet demands for online monitoring, quick, portable, easy to use, small amounts of chemical reagents, and cheap analysis. Based on this, electrochemical techniques meet these expectations and are recommended as an alternative due to their high sensitivity, good selectivity, portability, low cost, simple instrumentation, fast analysis, and easy to operate.^16–20^

In such kind of electrochemical techniques, working electrode materials are the main components that are employed as solid platforms for immobilization of recognition or sensing elements and regulate the charge as well as mass transfer.^20–22^ In that essence, numerous nanomaterials that provided synergic electrocatalytic properties along with expanding the working surface area, hence, loading capacity and mass transport of reactants were implemented for achieving high performance in terms of selectivity and sensitivity issues. In this regard, polyurethanes (PUs), the distinctive class of polymers which are widely used in construction materials, biomedical, actuators, and transducers, have been applied in the construction of electrochemical sensing platforms.^23–29^ Basically, PUs are synthesized by combining three chemical constituents (diisocyanate (aliphatic or aromatic), polyether diol (long-chain polyester), and diamine (small molecule chain-extender diol)).^27,30,31^ Modified poly(urethane urea) (PUU) provides high thermal and mechanical stability.^32^ Therefore, PUU has been implemented in sensing of gas, humidity, pressure, hormone, and glucose.^33–38^ In the electrochemical sensor sector, polyurethane composite decorated with gold nanoparticles has been used to detect dopamine in the cerebrospinal synthetic fluid.^39^ Also, polyurethane modified with gold nanoparticles has been used to determine tryptophan.^40^ Thus, the PUU was selected in this study as a polymeric platform for the voltammetric determination of Hg^2+^ ions in fish tissues. The PUU was prepared through the one-shot technique and then doped with different nanomaterials to enhance the PUU-electrochemical features. The PUU-based platforms were characterized by FTIR, TGA, DSC, X-ray diffraction, TEM, SEM-mapping, and cyclic voltammetry. Eventually, the modified electrodes with the PUU/Au NPs were finally selected as the targeted sensing platforms.

## Materials and methods

### Chemical reagents and substances

4,4′-Diphenylmethane-diisocyanate (MDI), castor oil (CO) and ethanolamine (EA) were purchased from Merck, Germany. Tin(ii) octoate, Sn(Oct)_2_ was used as a catalyst and it has been provided by Sigma-Aldrich. Tetrahydrofuran (THF) and *N*,*N*-dimethylformamide (DMF) were obtained from Fisher chemicals. Phthalic acid (C_8_H_6_O_4_) was used as a supporting electrolyte and prepared in Milli-Q water. The synthesized nanomaterials were used in this study include, silver (Ag), gold (Au), platinum (Pt), and copper (Cu) nanoparticles in addition to metal oxides such as aluminum oxide (Al_2_O_3_), manganese oxide (MnO_2_) nanorods, and multi-walled carbon nanotube (MWCNTs, Sigma-Aldrich). Carbon paste electrodes were prepared by using graphite powder and paraffin oil, brought from Acros Organics and Fluka, respectively.

### Chemical synthesis of poly(ester-urethane) urea (PUU)

**Scheme 1:**
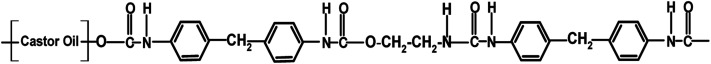
Chemical structure of the synthesized PUU.

As presented in Scheme 1, the PUU, including MDI, CO, EA, is chemically synthesized by one-shot poly-condensation technique as reported in our previous work.^27,41^ The solutions of MDI, CO, EA with molar ratios (1.0 : 0.5 : 0.5) were placed in 250 mL polypropylene beakers containing tin(ii)-2-ethylhexanoate (0.03 wt%, concerning to the reactants) and stirred vigorously. The resulting viscous polymer was then poured into a silica mold and heated at 50 °C for 100 h in an oven to attain a complete polymerization. Then, the samples were transmitted to a roll mill mixer for 15 minutes to eliminate any air bubbles through the casting process. Consequently, the sample was subjected to hot press, and compression molded into 1 mm plates at 175 °C for 45 min at a pressure of 62.05 MPa, and then cooled down to room temperature. The resulting PUU was dissolved in a mixture of tetrahydrofuran and *N*,*N* dimethylformamide (THF/DMF, 1 : 1 v/v).

### Preparation of gold nanoparticles (Au NPs)

Using a chemical reduction method, the synthesis of Au nanospheres was carried out as follows: a solution of chloroauric acid (HAuCl_4_·3H_2_O) with the concentration of 147 mM was prepared in 50 mL of Milli-Q water. The solution was heated until boiling in 100 mL glass flask. A volume of 2 mL of 1% (w/v) tri-sodium citrate was then added rapidly under vigorous stirring. The solution color turned from pale yellow to blue and eventually to dark red color. The suspension was then centrifuged for 15 min at 15 000 rpm, and re-suspended in water to be used for drop-casting on the electrode surface.

### Preparation of working electrode

For electrochemical characterization, the PUU was doped with different nanomaterials *via* forming a homogenous suspension which is then mixed with graphite powder (1 : 10 w/w) and paraffin oil to form a carbon paste. The resulting paste was then packed into a cylindrical plastic tube with 5 mm internal diameter. The surface of the modified electrodes was smoothed and polished with wet filter paper, and rinsed with Milli-Q water. Finally, prior to each electrochemical measurement, surfaces of the modified electrode were activated electrochemically by running continues voltammetric cycles from −0.4 to 1.0 V (6 voltammetric cycles) in KCl 0.1 M, using a scan rate of 50 mV s^−1^ and equilibrium time of 15 seconds.

### Measurements and devices

The voltammetric measurements were carried out using a computer-controlled Gamry potentiostat/galvanostat/ZRA G750 (Gamry, Pennsylvania, USA). The electrochemical system is connected with a three-electrode setup (the PUU-based carbon paste electrode as the working electrode, Ag/AgCl as a reference electrode, Pt disc as a counter electrode). Cyclic voltammetry (CV) technique was carried out in a 50 mL electrochemical cell containing phthalic acid (pH 2.5) as supporting electrolyte medium, at the potential window between −0.4 to 1.0 V and scan rate 50 mV s^−1^. Heavy metal ions were accumulated at the modified electrode surface for 15 seconds at the applied potential of 0.0 V).

The pH measurements were performed by using a bench-top pH-meter (JENWAY, Model 3510, UK). Fourier transform infrared (FTIR) spectra were obtained using Thermo Scientific IS10 FTIR spectrometer (USA). The FTIR spectra were collected in the range of 4000–400 cm^−1^ at room temperature, utilizing 64 scans at 4 cm^−1^ resolutions. For morphological characterizations, high-resolution transmission electron microscopy (HR-TEM, JEOL JEM-2100) at an accelerating voltage of 200 kV was used for imaging and analysis. Scanning Electron Microscope (SEM, JEOL, JXA-840A) at an accelerating applied potential of 15 keV. Differential Scanning Calorimetry (DSC), Q20 V24.10 Build 122 (USA), approximately 6 mg of a sample was measured, temperature range from 0 to 400 °C at a heating rate of 10 °C min^−1^, under nitrogen flow of 40 mL min^−1^. Thermogravimetric Analysis (TGA), (DTG-60H, Shimadzu, Japan). Approximately 8 mg of sample was measured from 0 to 500 °C at a rate of 10 °C min^−1^ under a nitrogen flow of 40 mL min^−1^. X-ray diffraction (XRD) analysis was performed using Shimadzu XRD6000 Japan, operating with nickel filtered, Cu–K target, voltage 40 kV, current 30 mA, and scan speed 8 degrees per min.

### Fish sample pre-treatment and mercury ions determination

Different fish samples and shrimps were brought from local markets. The muscle tissues of fishes and shrimps were removed, homogenized, and 1.0 gram of each dried tissue sample was transferred into a 25 mL digestion flask and 10 mL concentrated HNO_3_ (69–70%) was added. The flask was then heated at 40 °C for 1 hour and the temperature was then raised up to 150 °C for another 3 hours. Digestion was continued until all tissue samples dissolved completely in the conc. acid, *i.e.* continuous boiling of the acid–tissue mixture until dense white fumes appeared. Next, the digested samples were cooled and filtered through the Whatman No. 42 filter paper. The samples were diluted up to 5 mL of distilled water for analysis.^42^

## Results and discussion

### Synthesis and characterization of poly(ester-urethane) urea (PUU)

The PUU was prepared by one-shot polycondensation technique, three monomers (MDI, CO, EA) are synthesized, both CO and EA carry hydroxyl and amino groups to react with isocyanates MDI group to give urethane and urea groups localized on the same chain. The final polymeric structure provides urethane and urea groups that have the ability to bind with some heavy metals. Gold nanoparticles are introduced into the PUU to provide the electrochemical activity of the materials because of their good electrochemical behavior.

**Fig. 1:**
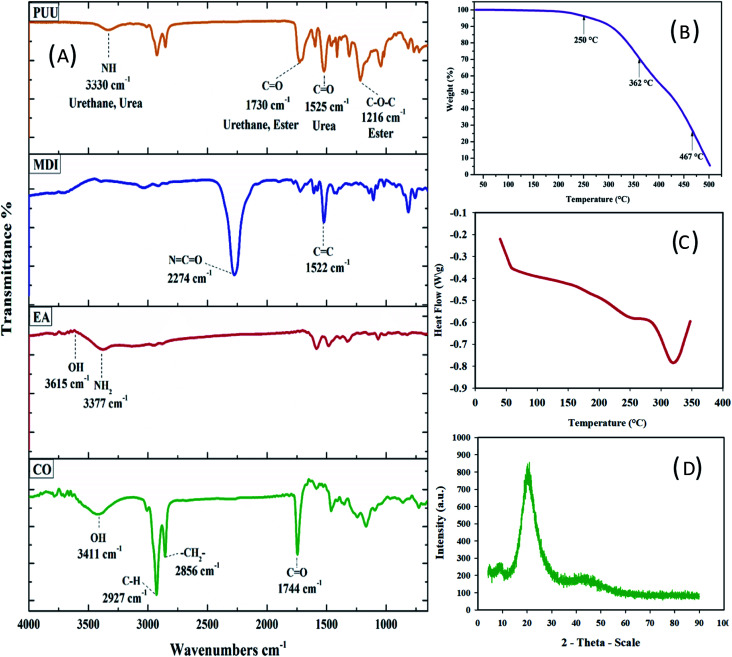
(A) FTIR spectra of CO, EA, MDI and PUU, and (B) TGA curve of PUU, (C) DSC thermogram of PUU, and (D) XRD diffractogram of PUU.

Before involving the PUU into electrochemical studies, a series of characterization techniques such as FTIR, TGA, DSC, X-ray diffraction, TEM, and the SEM were applied to analyze the chemical structure, thermal stability, and morphological properties of the synthesized PUU. Successful urethane-urea formation was confirmed by infrared spectra of MDI, EA, CO and PUU, and presented in Fig. 1A. In the spectrum of CO, the absorption peaks at 3411 cm^−1^, 2927 cm^−1^, 2856 cm^−1^, and 1744 cm^−1^ are ascribed to O–H stretching vibration, CH_2_ symmetric and asymmetric, and C=O ester group respectively. The absorption peaks at 3377 cm^−1^ and 3615 cm^−1^ are peaks ascribed to N–H/O–H groups of EA. A strong band at 2274 cm^−1^ is assigned to the NCO group of MDI^43,44^ and the stretching vibration peak of the benzene ring C=C at 1522 cm^−1^. In the spectra of the final PUU product, the absorption peak at 3330 cm^−1^ that corresponds to N–H group and the characteristic absorption peak at 2274 cm^−1^ that corresponds to the isocyanate of MDI were disappeared. Furthermore, the newly formed peaks at 1730 and 1525 cm^−1^ are attributed to the stretching vibration of –NHCOO– and NHCONH– groups, indicating that the MDI, CO and EA are completely reacted and the PUU was successfully synthesized.

The existence of PUU thermal stability, is referred to as including the raw materials, *i.e.* proportions of soft and hard segments, density and type of crosslinking bonds, type of chain extender and the synthesis routes.^45^ Here, the thermal stability was evaluated by TGA and the results are given in Fig. 1B. Three stages of the thermal decomposition were observed in the TGA-thermogram, where the first stage of weight loss was around 250 °C, is probably due to the vaporization of volatilized products/solvent that might be encapsulated within the PUU chain. The second stage has a maximum degradation rate at 362 °C, involving the complete decomposition of urethane and urea bonds in rigid segments. While the third stage was obtained at 467 °C which is attributed to the thermal decomposition of ester bonds in soft segments. Regarding the glass transition temperature and melting behavior, the DSC analysis showed the glass transition temperature (*T*_g_) at 59 °C and, the melting temperature (*T*_m_) is 456 °C, as depicted in Fig. 1C.

On the other hand, X-ray diffraction analysis (XRD) was carried out to determine the crystalline phase of the prepared PUU. In the diffractograms, two broad diffraction peaks, one large and one small, were observed at 2*θ* = 21° and 9°, respectively. The two diffraction patterns displayed amorphous nature and did not exhibit crystalline peaks of the PUU Fig. 1D. The amorphous phase of PUU depends upon its structure and the presence of urea groups.

### Electrochemical characterization of PUU nanocomposites

**Fig. 2:**
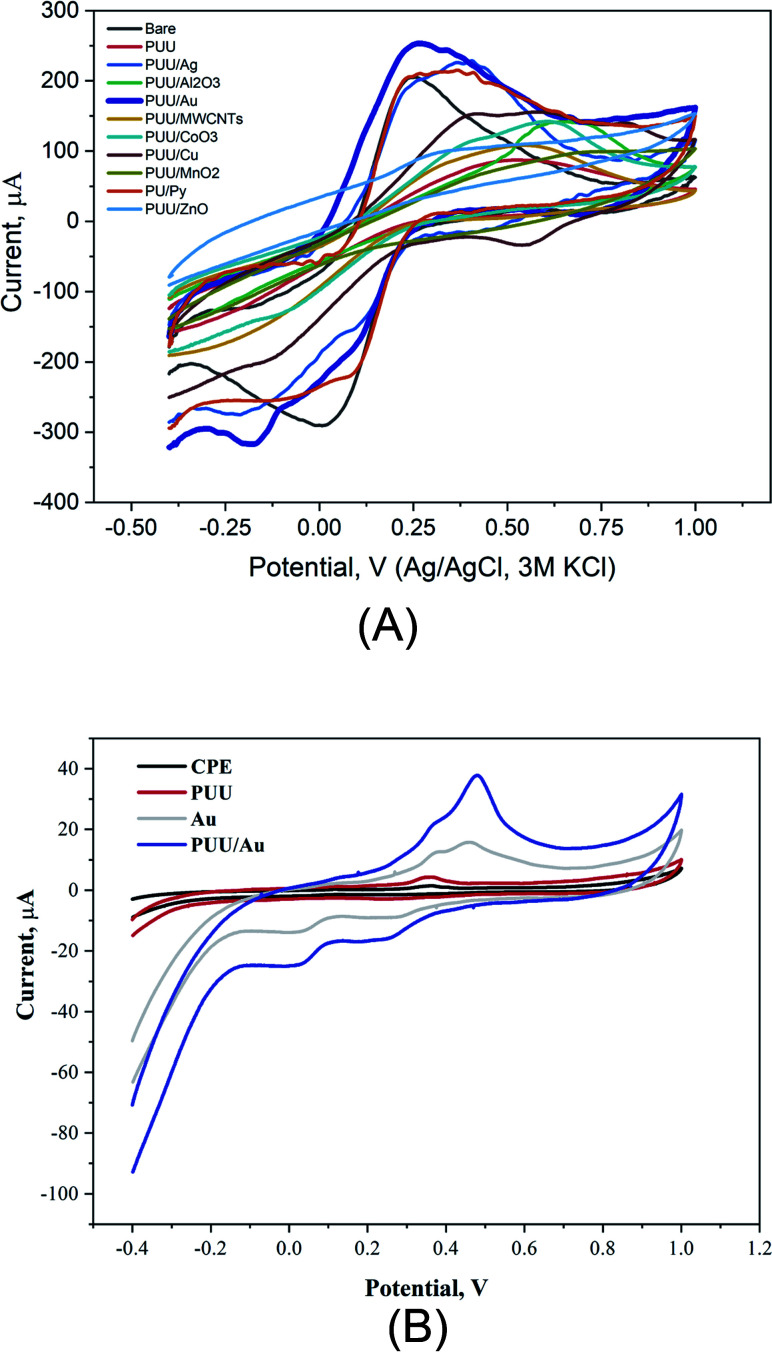
(A) Cyclic voltammetric measurements of FCN redox reactions at the surfaces of the bare, PUU, and PUU-different nanocomposites in the solution KCl containing 1 mM [Fe(CN)_6_]^3−/4−^. CVs were conducted in the potential range of −0.4 V to 1.0 V *vs.* the Ag/AgCl (3 M KCl), and the scan rate was 50 mV s. (B) Voltammetric responses of CPEs modified with PUU, AuNPs, and PUU/Au nanocomposite in compare with the bare electrode. The Hg^2+^ concentration in the electrochemical cell was 0.4 μg mL^−1^, scan rate was 50 mV s^−1^. The PUU, and the AuNPs concentrations in the electrode matrix were 4 and 6%, respectively.

In electrochemical systems, studying electron transfer rate at the electrode/solution interface provides information to understand the role of electrode composition(s) in the studied electrochemical process.^46–49^ Here, using cyclic voltammetry, redox reactions of ferricyanide, as a standard redox probe was studied at the modified electrodes with the PUU and PUU-nanocomposites (*i.e.* the PUU doped with each of these nanostructures: (Ag, Au, Pt, Cu, ZnO, CoO_3_, MnO_2_, Al_2_O_3_, and MWCNTs). As a result, a significant difference between electrochemical signals obtained by the PUU alone and that obtained by the PUU nanocomposites, as shown in Fig. 2A. On the other hand, voltammetric signals of Hg ions at the modified electrodes with AuNPs or PUU/Au were much higher than that obtained by the bare or the PUU based surfaces Fig. 2B. Thus, the use of PUU/Au is promising in the voltammetric recognition of Hg ions. Thus, PUU/Au nanoparticles have been assigned for the voltammetric determination of Hg^2+^ under further optimization.

### Morphologic characterization of PUU and PUU-Au

**Fig. 3:**
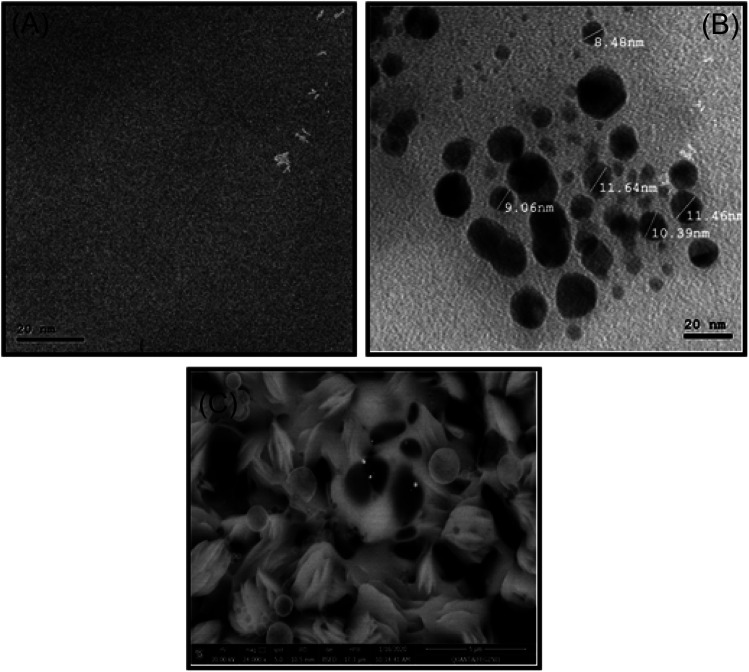
(A) TEM image of the PUU morphology, (B) TEM image of the PUU/Au nanocomposite morphology, and (C) SEM image of the PUU/Au nanocomposite-3D structure.

The PUU/Au nanocomposite is being selected as an electrode modifier for the detection of Hg ions, therefore, morphological characterization of the nanocomposite constituents (*i.e.* the PUU or the PUU/Au) were performed. From the TEM images, a compact and smooth surface of the PUU was observed Fig. 3A. On the other side, a correlation between the TEM and the 3D-SEM images Fig. 3B and C revealed that the PUU structure is modified non-covalently with spherical particles sized with 10 nm that are composed of the AuNPs.

### Assay optimization

#### Effect of electrode composition

**Fig. 4:**
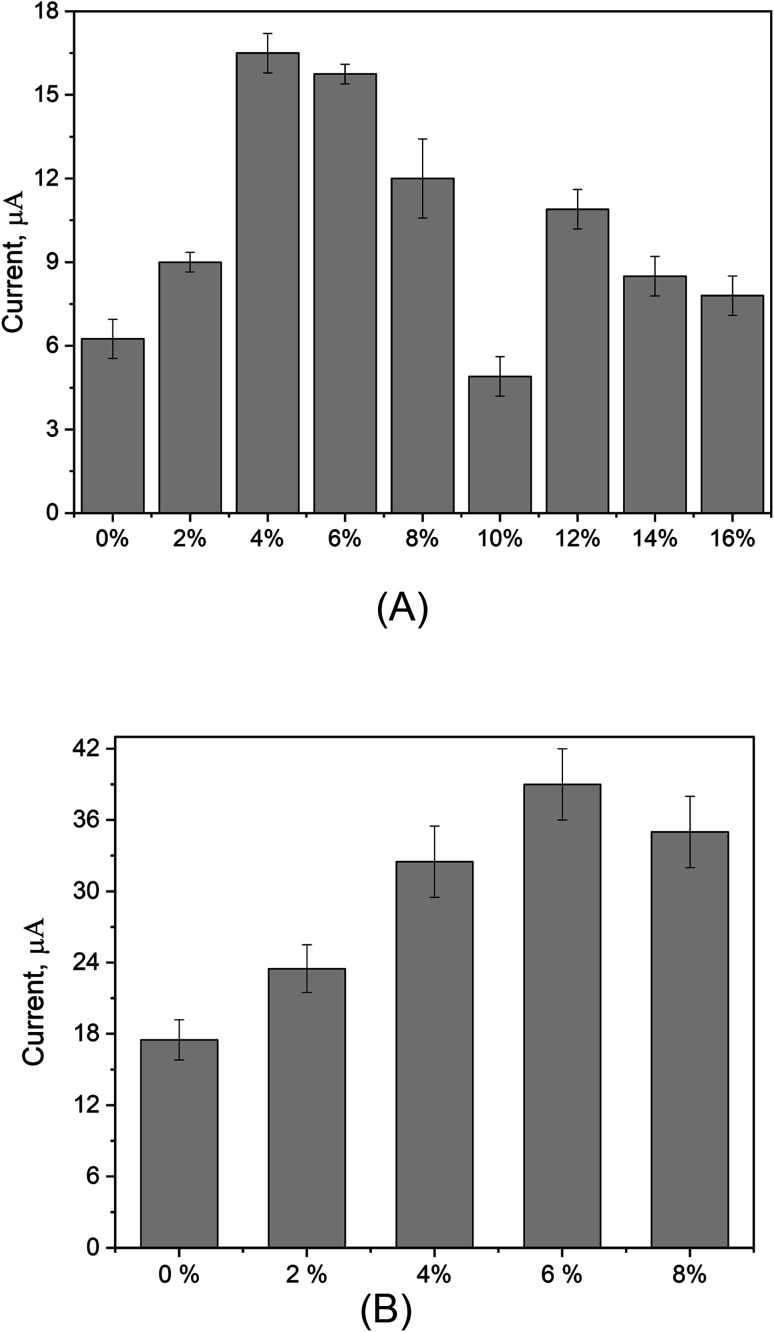
(A) Voltammetric responses of modified electrodes with different concentrations of PUU within the electrode matrix. The presented current values are the Δ*I*_oxid_ (oxidation peak current of mercury subtracted from the baseline of the electrolyte). The AuNPs concentration was fixed at 8%. (B) Voltammetric responses of the chemically modified electrodes with different AuNPs concentrations towards the mercuric oxidation. The presented current values are the Δ*I*_oxid_ (oxidation peak current of mercury subtracted from the baseline of the electrolyte). The PUU concentration was fixed at 4%.

The modified electrode composed of the PUU/Au is consigned for the voltammetric recognition of mercury(ii) ions, thus the effect of electrode composition (variation of PUU and AuNPs concentrations in electrode matrix) on the re-oxidation of Hg^0^ at the electrode surface is investigated. In this regard, carbon paste electrodes were made of a wide range of PUU concentrations (ranging from 0 to 16% out of the total electrode composition). As a result, PUU concentration dependency was observed from the voltammetric signals, whereas the oxidation peak currents were remarkably increased until the 4% of PUU and then a decrease in the voltammetric signals was obtained due to the increase of PUU concentrations in the paste composition, as shown in Fig. 4A. In the same manner, the increase of oxidation current was dependent on the concentration of gold nanoparticles within the electrode matrix until the Au NPs reached 6%. A decrease in the oxidation current was obtained above this concentration, as shown in Fig. 4B. Thus, 4% and 6% were chosen as optimal concentrations for PUU and Au NPs, respectively.

#### Effect of pH

**Fig. 5:**
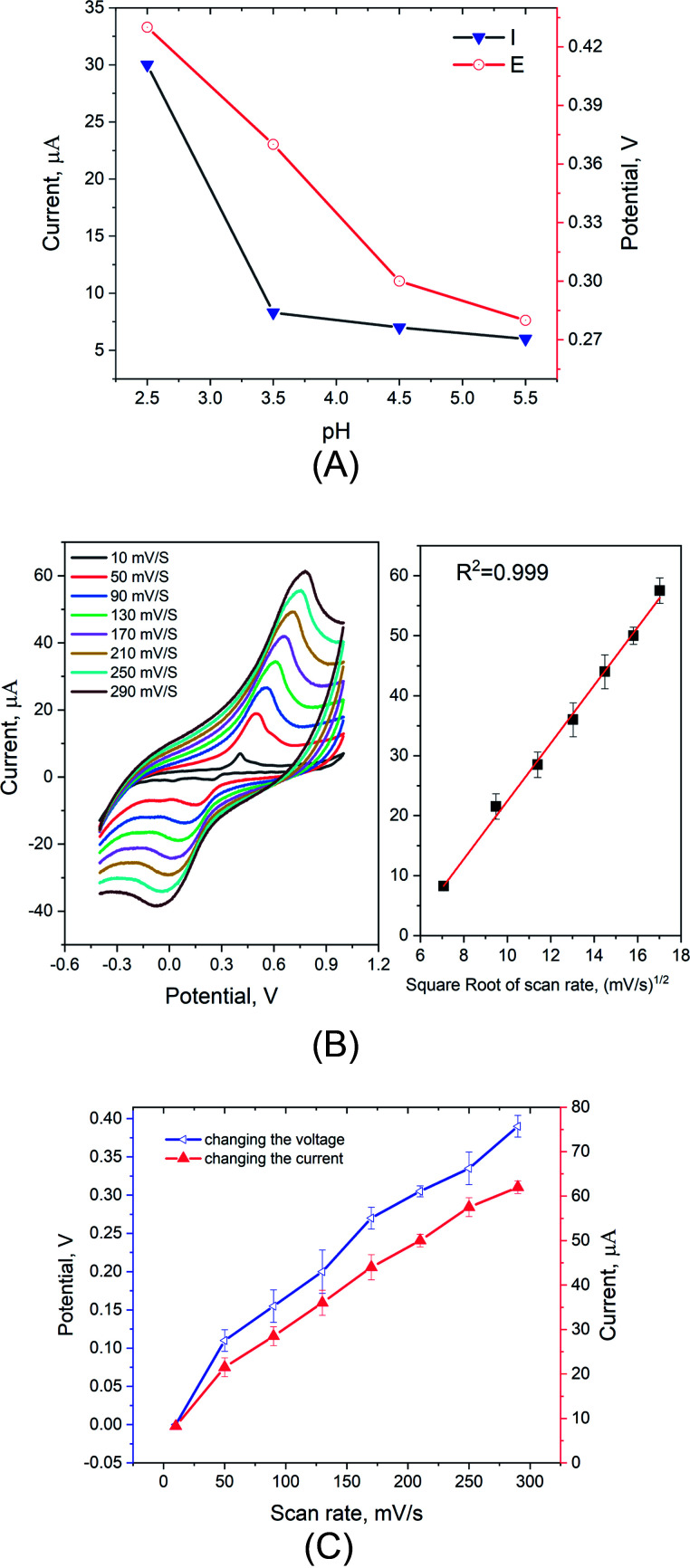
(A) Effect of pH on the voltammetric responses (peak potential and oxidation current) of the PUU-AuNPs-modified electrode towards 0.4 μg mL^−1^ of Hg^2+^. Scan rate 50 mV s^−1^ and 15 s for the equilibrium time. (B) Effect of scan rate on the changes of peak currents and peak positions. (C) Effect of scan rate on the changes of peak currents and peak positions.

The influence of pH on the electrode performance was assessed by measuring the voltammetric signals (changes of the oxidation current of mercury ions over the changes of the peak potential position). As shown in Fig. 5A, increasing the pH of the supporting electrolyte causing a lowering of the oxidation peak height. On the other hand, the associated anodic peak potential (*E*_pa_) in CV shifted from positive to negative potential. This shift in the peak potential might be attributed to the decrease in the number (concentration) of the mercuric ions accumulated at the electrode surface which is directly linked to the diffusion controlled reaction whereas the double layer is very large at higher potential (*i.e.* higher pH of the supporting electrolyte).^48^ This explanation is correlated with the decreasing of the oxidation peak heights. Thus, the chemical structure of the PUU/Au is sensitive to the pH changes when it comes to interact with mercuric ions.

#### Effect of scan rate

Referring to the results obtained by the effect of pH on the peak current and peak potential, the surface-controlled process is characterized here by studying the influence of voltammetric scan rate on the rate of mercuric ions transport from the bulk to the PUU/Au interface. Fig. 5B showed that, at fixed concentration of Hg^2+^, the voltammetric peaks were increasing linearly with the increase of scan rate, a CV (from 10 to 290 mV s^−1^). As has been described by Randles–Sevcik equation,^50^ the peak current was proportional to the square root of the scan rate due to the fast electron transfer process with diffusion limited. In addition to the change of peak heights, the peak (position) potential was shifted towards positive voltage direct due to the increase in the scan speed Fig. 5C. Hence, from studying and analyzing the effect of scan rate, we conclude that the interaction of PUU/Au with the targeted ions, is a diffusion-controlled process, which is correlated with that obtained by the pH effect.

#### Accumulation time

**Fig. 6:**
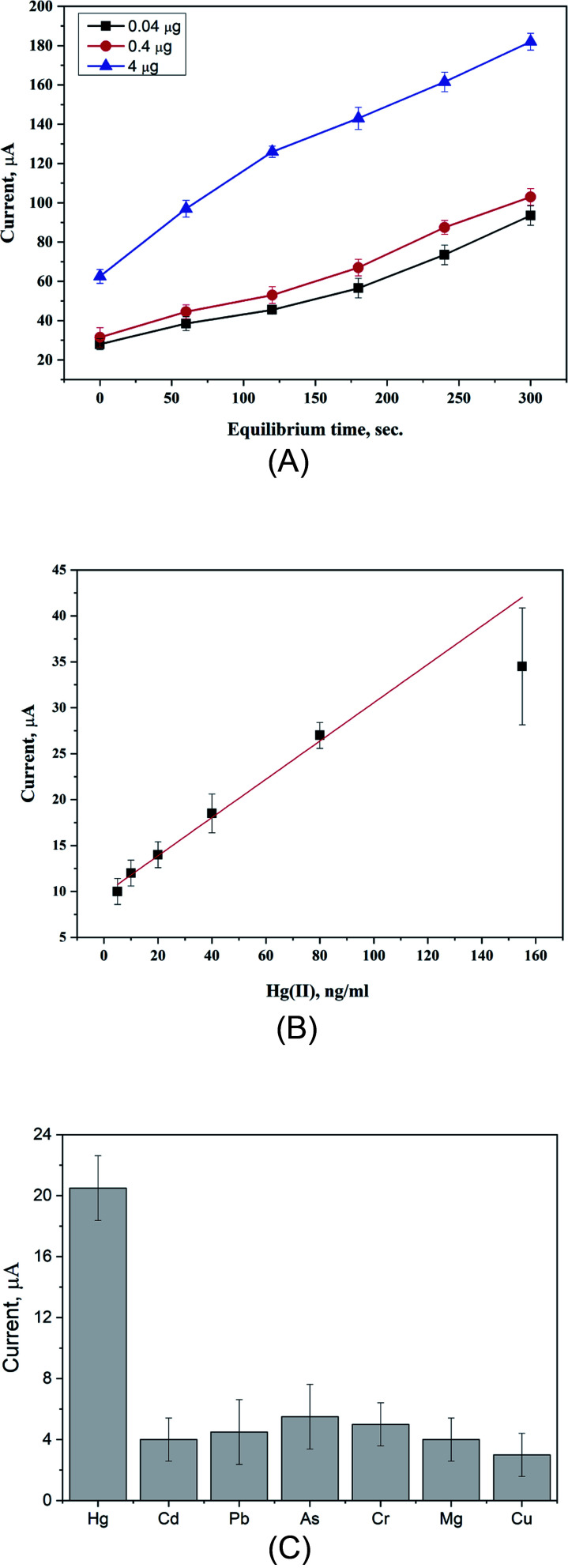
(A) Effect of accumulation time on the oxidation current of the target mercury ions using the PUU/Au-based electrode. (B) Calibration curve for the voltammetric determination of Hg^2+^ under optimal conditions. The error bar is the standard error from three independent measurements. The calculated probability value (*P* < 0.0001). (C) Voltammetric responses of the PUU/Au-based electrode towards the detection of the target analyte and other interferents substances. The experiments were conducted 0.4 μg mL^−1^ of each heavy metal ions.

The binding capacity of the proposed sensor's surface to the target ions was tested using the accumulation time effect, whereas several accumulation time intervals (from 0 to 300 s) were applied while the concentration of mercuric ions was fixed. Then, the generated voltammetric signals were analyzed and the process is repeated on two other higher mercuric concentrations, as shown in Fig. 6A. As a result, a continuous increase in the oxidation peak current was obtained along with increasing the accumulation times. This coincided with all used concentrations (0.04, 0.4, and 4 μg mL^−1^ of Hg^2+^). Even at quite high concentration of mercury ions, the peak height was not dropped at the long duration of accumulation, *i.e.* no surface saturation was reached. From this finding, we can conclude that the functionalized electrode surface has a high capacity towards the accumulation of high concentration of the target ions and this is attributed to the expanded surface area as a nanocomposite structure.

#### The principle of the Hg^2+^ detection

**Scheme 2:**
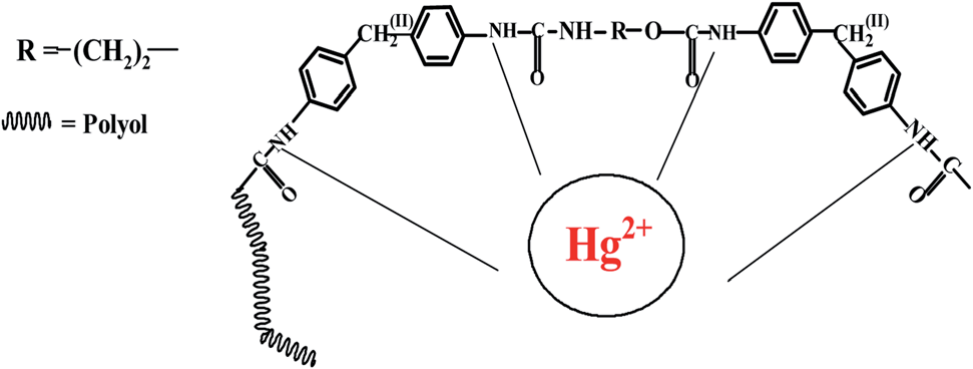
Sensing scheme of Hg ion using PUU based electrode.

As a polymeric structure, the PUU consists of urethane and urea groups with functions that possess pronounced binding abilities to Hg^2+^ ions Scheme 2. To confirm the interaction mechanism of the PUU-based sensor with Hg^2+^, FTIR spectroscopy was carried out Fig. S1, ESI,† whereas after incubating Hg^2+^ with the modified surfaces, the N–H stretches of amines at 3334 cm^−1^ was more intense than that of untreated with Hg^2+^. The results revealed that N–H, linked to the Hg^2+^*via* a dative covalent bond. SEM-EDS mapping images combined with Energy Dispersive X-Ray Analysis (EDX for the elemental surface analysis) were conducted with the PUU/Au electrode incubated with Hg. Well distribution, and fully covered surface with Hg were detected, as shown in Fig. S2A, ESI.† EDX spectra Fig. S2B, ESI† also showed peaks corresponding to (Hg, Au, C, N, and O) which are the elemental composition of the electrode surface that captured the mercury ions.

#### Calibration curve

Under optimal conditions, the anodic peak currents were obtained for a series of standard solutions of Hg^2+^, whereas a calibration curve of peak current *versus* concentration was constructed. As a result, in Fig. 6B, a linear relationship was obtained over the concentration range (5 ng mL^−1^ to 155 ng mL^−1^) with the regression coefficient (*R*^2^ = 0.986), while the limit of detection and limit of quantification were 0.235 ng mL^−1^, and 0.710 ng mL^−1^ respectively. Worth mentioning here that the proposed method is showing a higher sensitivity over the other previously published voltammetric method for mercuric ion detection, as shown in Table 1.

**Table 1:** Selected published recent reports for Hg(ii) determination using different modified electrodes by electrochemical techniques

Sensing platform	Technique	Detection limit	Linear range	Ref.
PPh3/MWCNTs/IL/CPE	SWASV	9.2 × 10^−5^ μM	1 × 10^−4^–0.15 μM	51
AuNPs/CFME	DPASV	0.1 μM	0.2–50 μM	52
12-crown-4-ether/MWCNTs	LSV	1.25 μM	25 μM–550 μM	18
Functionalized gold nanoparticles/reduced graphene oxide	DPV	7.5 nM	50 nM–5 μM	53
Au/DMAET/(SWCNT-PABS)	SWASV	0.06 μM	20–250 μM	54
Mg–Al–TGA LDH/GCE	SWASV	0.8 nM	2.0–800 nM	55
EDTA/CPE	SWV	16.6 × 10^−9^ M	5 × 10^−5^–35 × 10^−5^ M	56
CuO/PVA/GCE	CV	0.42 nM.	10–70 μM	57
2,2′-(Ethane-1,2-diylbis((2-(azulen-2-ylamino)-2-oxoethyl) azanediyl))diacetic acid	DPV	3 × 10^−10^ M	10^−9^–5 × 10^−7^ M	58
NRGO/GCE	DPASV	0.58 nM	1 nM to 800 nM	59
Thiosemicarbazone ligands	DPV	7 × 10^−10^ M	10^−9^–10^−6^ M	60
ssDNA-cysteine/gold electrode surface.	SWV	10 (pM)	100 (nM) −10 (pM)	61
AuNPs – GC	(SWASV)	0.42 nM	0.64–4.00 nM	62
Poly(aniline-*co-o*-aminophenol) – PANOA/Au NPs	ASV	0.23 nM	0.8–12.0 nM	63
PUU-Au/CPE	CV	1.17 nM	25 nM–775 nM	*This work

#### Effects of interferents

For the selectivity point of view, the performance of the PUU/Au-based electrode was tested against other heavy metal ions such as Cd^2+^, Pb^2+^, As^3+^, Cr^3+^, Mg^2+^, and Cu^2+^. The experiments were performed individually in 0.03 M phthalic acid (pH 2.5) containing each a single concentration of the non-targeted metal ion (0.4 μg mL^−1^ for each). As shown in Fig. 6C, the Hg^2+^ were detected with a low significant overlap with the other metal ions. Therefore, the proposed sensor has a good selectivity for the detection of target analyte in real samples contaminated with the other heavy metals.

#### Reproducibility and repeatability testing

Five fresh prepared PUU/Au modified electrodes were prepared and utilized for their reproducibility testing towards the detection of mercury. Very similar behavior was obtained for all electrodes, since the baseline, the oxidation peak height, and peak potential that were obtained by each electrode were very much alike to each other. Statistically, with 95%, a very satisfied reproducibility of the proposed method was achieved. On the other hand, high repeatability (97%) was obtained when one modified electrode was used several times for detecting mercury through several trails (*i.e.* individual voltammetric measurements) using a single concentration of mercury (160 ng mL^−1^ for each). The performed reproducibility and repeatability tests showed the high stability and reproducibility of the modified electrodes towards the rapid tracking of mercury.

#### Detection of mercury in real samples

Determination of total mercury in fish tissues using the proposed voltammetric method was performed after spiking the fish and shrimp samples with standard mercuric concentration, followed by acid digestion. In this regard, eight fish tissue types (names and sources are listed in Table 2) were analyzed. The quick analysis with a good recovery rate (90% to 111%) occurred.

**Table 2:** Total mercury determination in fish tissues using the proposed voltammetric method

Sample	Spiked conc. (μg mL^−1^)	Detected conc. (μg mL^−1^)	Proposed method-recovery (%)
Tilapia-river Nile	4	3.7	93
Tilapia fish farming	4	4	100
Catfish-river Nile	4	3.84	96
Catfish-fish farming	4	4.4	111
Shrimp-Red Sea	4	3.57	90
Shrimp-fish farming	4	4.7	117
Tuna	4	3.78	95
Salmon	4	3.82	96

## Conclusion

Exploiting poly(ester-urethane) urea (PUU) as a new sensing platform for the rapid detection of mercury ions in fish samples was achieved. PUU incorporation with other nano-structures such as gold nanoparticles improved the electrochemical properties and the sensing responses reaching a very low limit of detection. Physicochemical characterizations were completely performed to define the physical and chemical properties, along with the surface morphology and sensing mechanisms. Eventually, assay optimization and real samples analysis using the new electrode were conducted. This new approach is directed to support the use of polymeric hetero-structures for the rapid tracking of hazardous in environmental and biological specimens.

## Author contributions

Hany Abd El-Raheem: methodology, validation, writing – original draft. Rabeay Y. A. Hassan: conceptualization, supervision, writing and review the manuscript. Rehab Khaled: review the manuscript. Ahmed Farghali: review the manuscript. Ibrahim M. El-Sherbiny: conceptualization, supervision, writing and review the manuscript.

## Conflicts of interest

The authors confirm that there are no conflicts to declare.

## Supplementary Material

RA-011-D1RA03693A-s001
