# Supplementary material for: New sensing platform of poly(ester-urethane)urea doped with gold nanoparticles for rapid detection of mercury ions in fish tissue
Source: RSC Adv. 2021 Sep 28;11(50):31845–54. doi: 10.1039/d1ra03693a (PMC9041571; doi:10.1039/d1ra03693a)
Supplement: RA-011-D1RA03693A-s001 [file RA-011-D1RA03693A-s001.pdf]

Electronic Supplementary Material (ESI) for RSC Advances.
This journal is © The Royal Society of Chemistry 2021

New sensing platform of poly(ester-urethane) urea doped with gold nanoparticles for rapid detection of mercury ions in fish tissue

Hany Abd El-Raheem[1,3], Rabeay Y. A. Hassan[1,2*], Rehab Khaled[4], Ahmed Farghali[3], Ibrahim M. El-Sherbiny[1*]

[1]Center of Materials Sciences, Zewail City of Science and Technology, October Gardens, 6th of October City, 12578, Giza, Egypt; [2]Applied Organic Chemistry Department, National Research Centre (NRC), Dokki, 12622, Giza, Egypt; [3]Materials Science and Nanotechnology Department, Faculty of Postgraduate Studies for Advanced Sciences, Beni-Suef University, Beni-Suef, Egypt; [4]Chemistry Department, Faculty of Science, Beni-Suef University, Beni-Suef, Egypt.

***Corresponding author:**
Prof. Ibrahim M. El-Sherbiny,
Center of Materials Sciences,
Zewail City of Science and Technology,
October Gardens, 6th of October City, 12578, Giza, Egypt
Email: ielsherbiny@zewailcity.edu.eg

***Corresponding author:**
Dr. Rabeay Y. A. Hassan, PhD
Zewail City of Science and Technology,
October Gardens, 6th of October City, 12578, Giza, Egypt
Email: ryounes@zewailcity.edu.eg

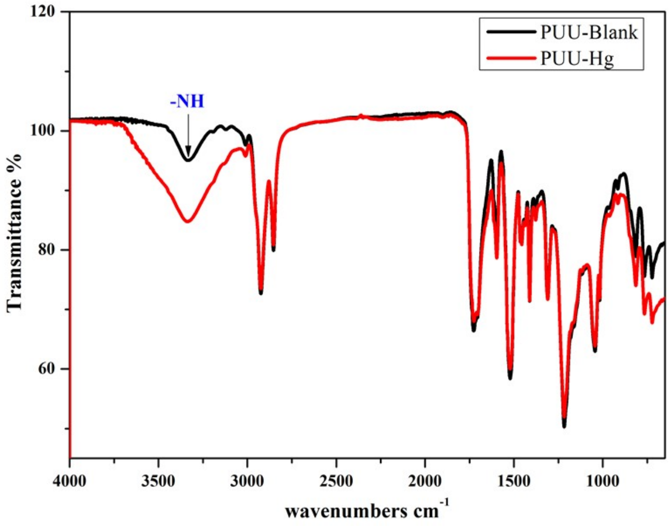

Figure-S1: FTIR spectra of PUU (black) before and after (red) interaction with mercury ion

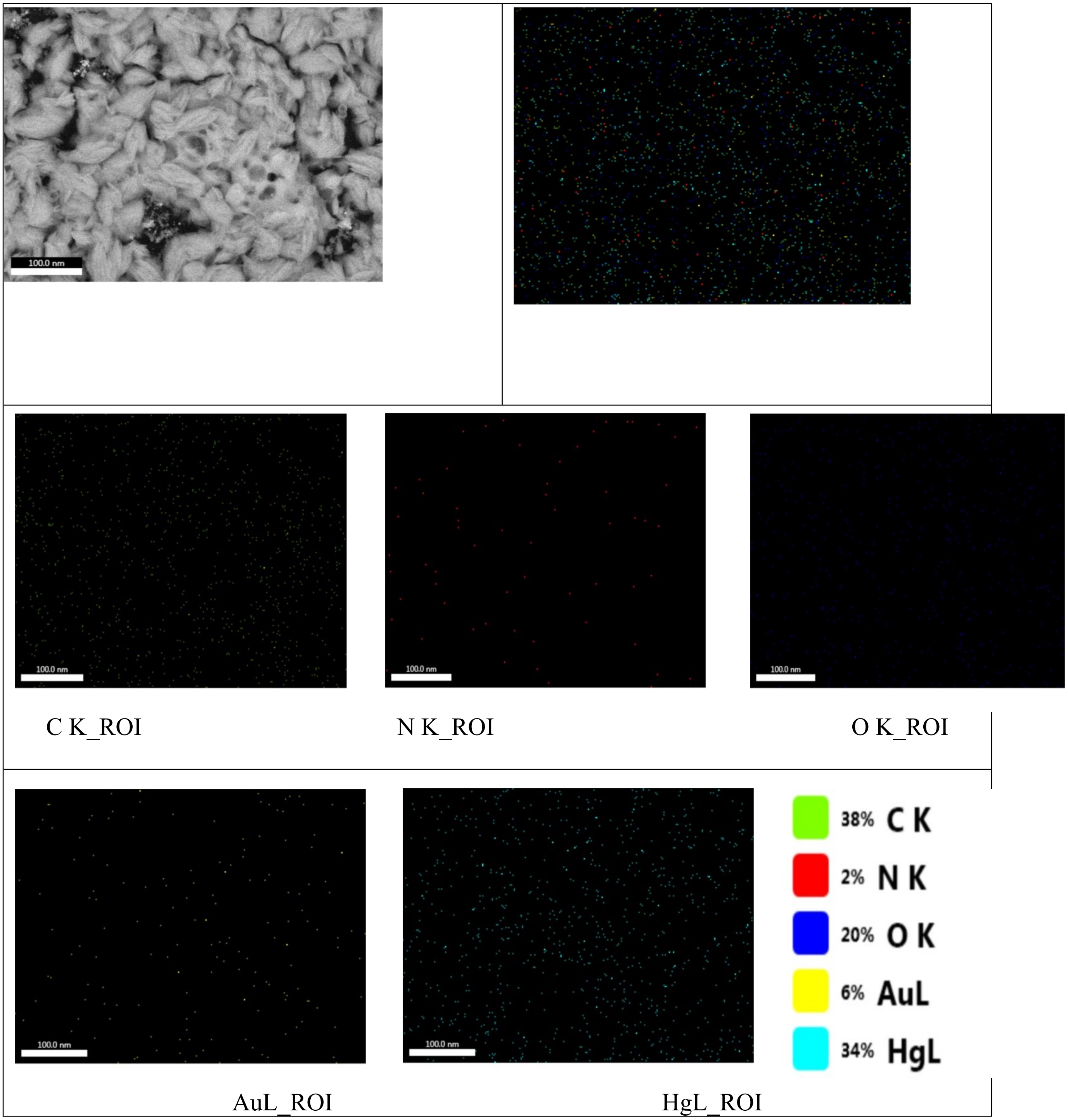

Figure-S2A: SEM-EDS mapping images of (Hg, Au, C, N, and O)

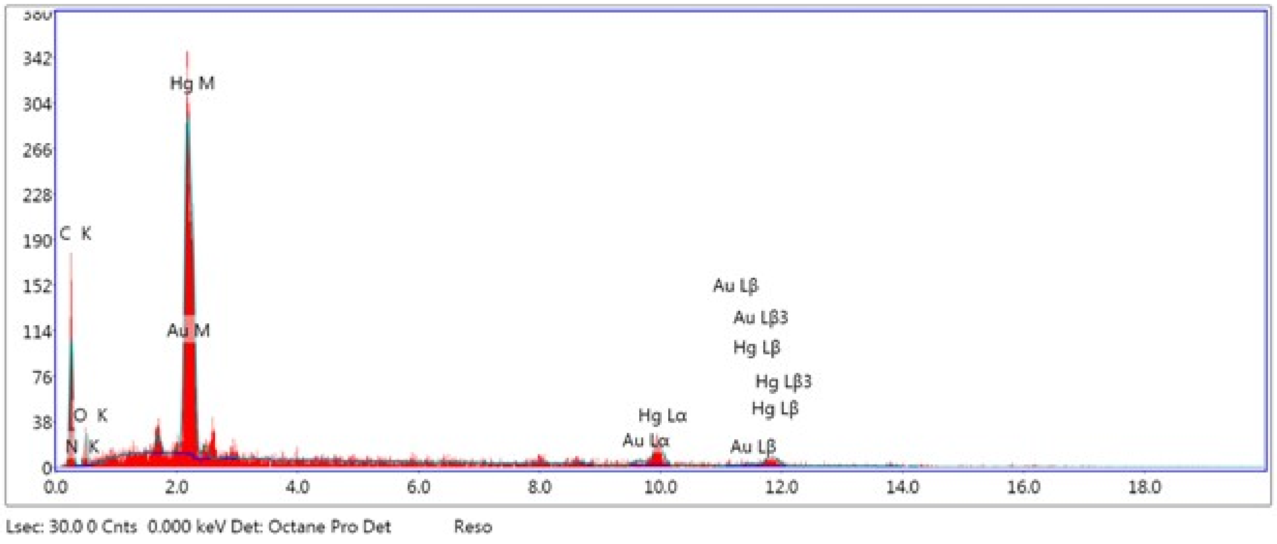

Figure-S2B: EDX spectra of (Hg, Au, C, N, and O)
